# Supplementary material for: Preclinical Studies with Glioblastoma Brain Organoid Co-Cultures Show Efficient 5-ALA Photodynamic Therapy
Source: Cells. 2023 Apr 10;12(8):1125. doi: 10.3390/cells12081125 (PMC10137158; doi:10.3390/cells12081125)
Supplement: Supplementary file 1 [file cells-12-01125-s001.zip › cells-2286384-supplementary.pdf]

cells

MDPI

Supplementary Material

Preclinical Studies with Glioblastoma Brain Organoid Co-Cultures Show Efficient 5-ALA Photodynamic Therapy

Leire Pedrosa [1,†], Carmen Bedia [2,†], Diouldé Diao [1], Alejandra Mosteiro [3], Abel Ferrés [3], Elisabetta Stanzani [4], Fina Martínez-Soler [5], Avelina Tortosa [5], Estela Pineda [6], Iban Aldecoa [7,8], Marc Centellas [9], Marta Muñoz-Tudurí [9], Ana Sevilla [10,11,*], Àngels Sierra [1,12,*] and José Juan González Sánchez [1,3]

1 Laboratory of Experimental Oncological Neurosurgery, Neurosurgery Service, Hospital Clinic de Barcelona—FCRB, 08036 Barcelona, Spain
2 Institute of Environmental Assessment and Water Research (IDAEA-CSIC), 08034 Barcelona, Spain
3 Department of Neurosurgery, Hospital Clínic de Barcelona, University of Barcelona, 08036 Barcelona, Spain
4 Laboratory of Pharmacology and Brain Pathology, IRCCS Humanitas Research Hospital, 20089 Milan, Italy
5 Apoptosis and Cancer Unit, Department of Basic Nursing, IDIBELL, Faculty of Medicine and Health Sciences, University of Barcelona, 08907 L'Hospitalet del Llobregat, Spain
6 Medical Oncology Department, Hospital Clinic and Translational Genomics and Targeted Therapies in Solid Tumors, IDIBAPS, 08036 Barcelona, Spain
7 Department of Pathology, Biomedical Diagnostic Center, Hospital Clínic of Barcelona, University of Barcelona, 08036 Barcelona, Spain
8 Neurological Tissue Bank of the Biobank, Institute of Biomedical Research August Pi i Sunyer (IDIBAPS), 08036 Barcelona, Spain
9 Laboratorios Gebro Pharma S.A., 08022 Barcelona, Spain
10 Department of Cell Biology, Physiology and Immunology, Faculty of Biology, University of Barcelona, 08028 Barcelona, Spain
11 Institute of Biomedicine, University of Barcelona (IBUB), 08036 Barcelona, Spain
12 Department of Medicine and Life Sciences (MELIS), Faculty of Health and Live Sciences, Universitat Pompeu Fabra, 08036 Barcelona, Spain
* Correspondence: anasevilla@ub.edu (A.S.); masierra@clinic.cat (À.S.)
† These authors have contributed equally to this work.

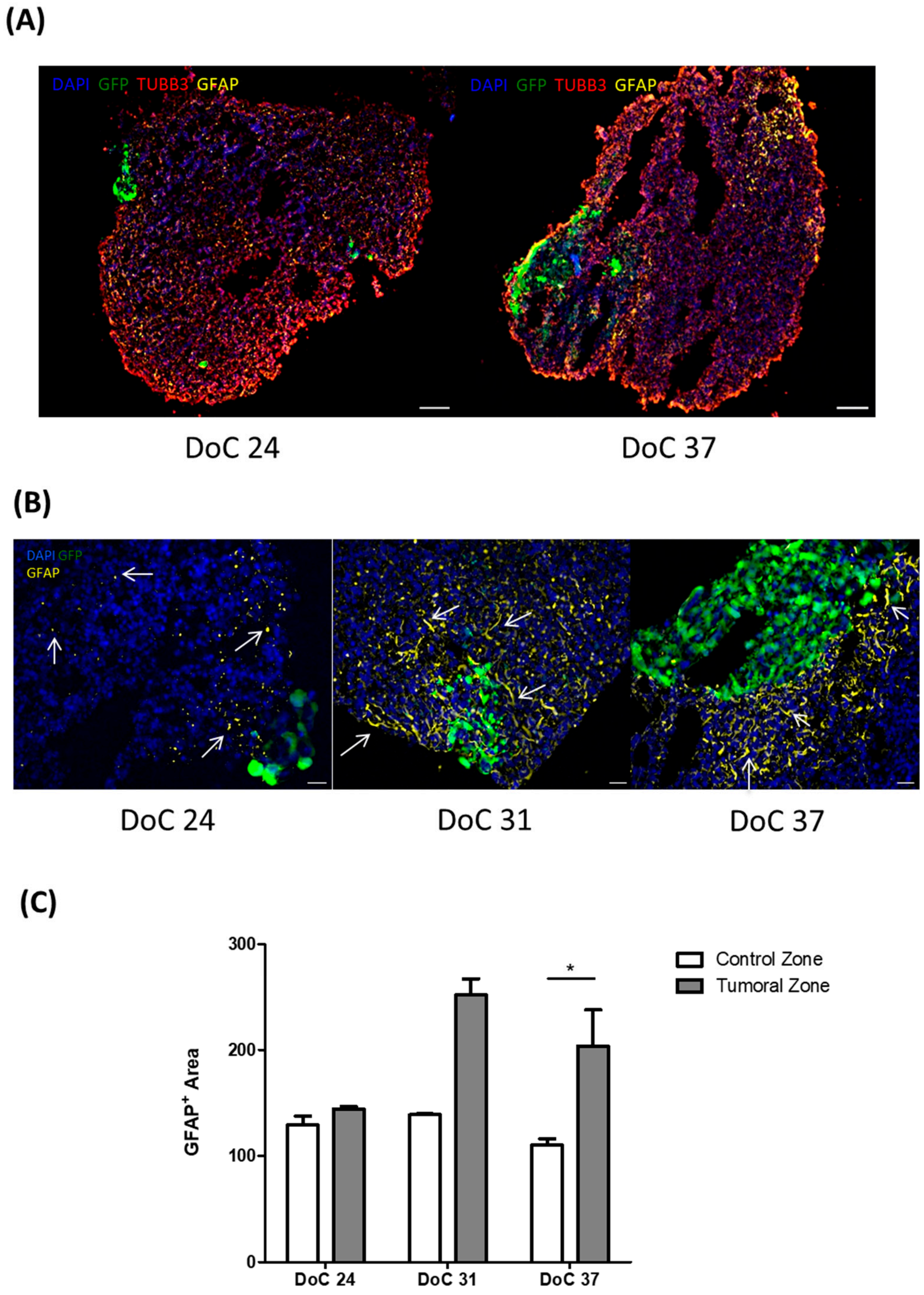

Figure S1. Immunofluorescence (IF) analyses of GFP-PG88-co-cultured with the cerebral organoid. (**A**) IF merged TUJ1 (red) and GFAP (yellow) at 24 (left) and 37 (right) days of co-culture (DoC). TUJ1 is homogenously expressed in all organoids on both days. *N* = 3. (**B**) Sequential images of samples on DoC 24 (left panel), on DoC 31 (middle panel), and on DoC 37 (right panel) after engraftment with GFP-PG88. Anti-GFAP antibody stain visualizing astrocytes in the human organoids are merged with GFP$^+$ cells signal and nuclei staining. Images are representative images of 3 different organoids analyzed, 6 fields with tumor and 9 fields without tumor, are obtained with the objective 10× and 40× with Microscopy Olympus BX41. Scale bar 100 µm (10×) and 20 µm (40×). White arrows highlight the GFAP$^+$ cells. **(C)** GFAP$^+$-cells area was studied in the tumor and control area far from the tumor-induced with PG88 cells at different days of organoid and GFP-GIC co-culture. The histogram represents the area of the GFAP$^+$ cells of organoid and GFP-GIC co-culture: day 24 (*n* = 3, 6 fields with tumor and 9 fields without tumor), day 31 (*n* = 2, 4 fields with tumor and 4 fields without tumor) and day 37 (*n* = 3, 8 fields with tumor and 6 fields without tumor). The GFAP

area was analyzed, by ImageJ, in GFP-GICs tumor area, and in areas without infiltrating GFP-GICs (control zone). ANOVA test was used to compare the means of different groups (* $p < 0.05$).

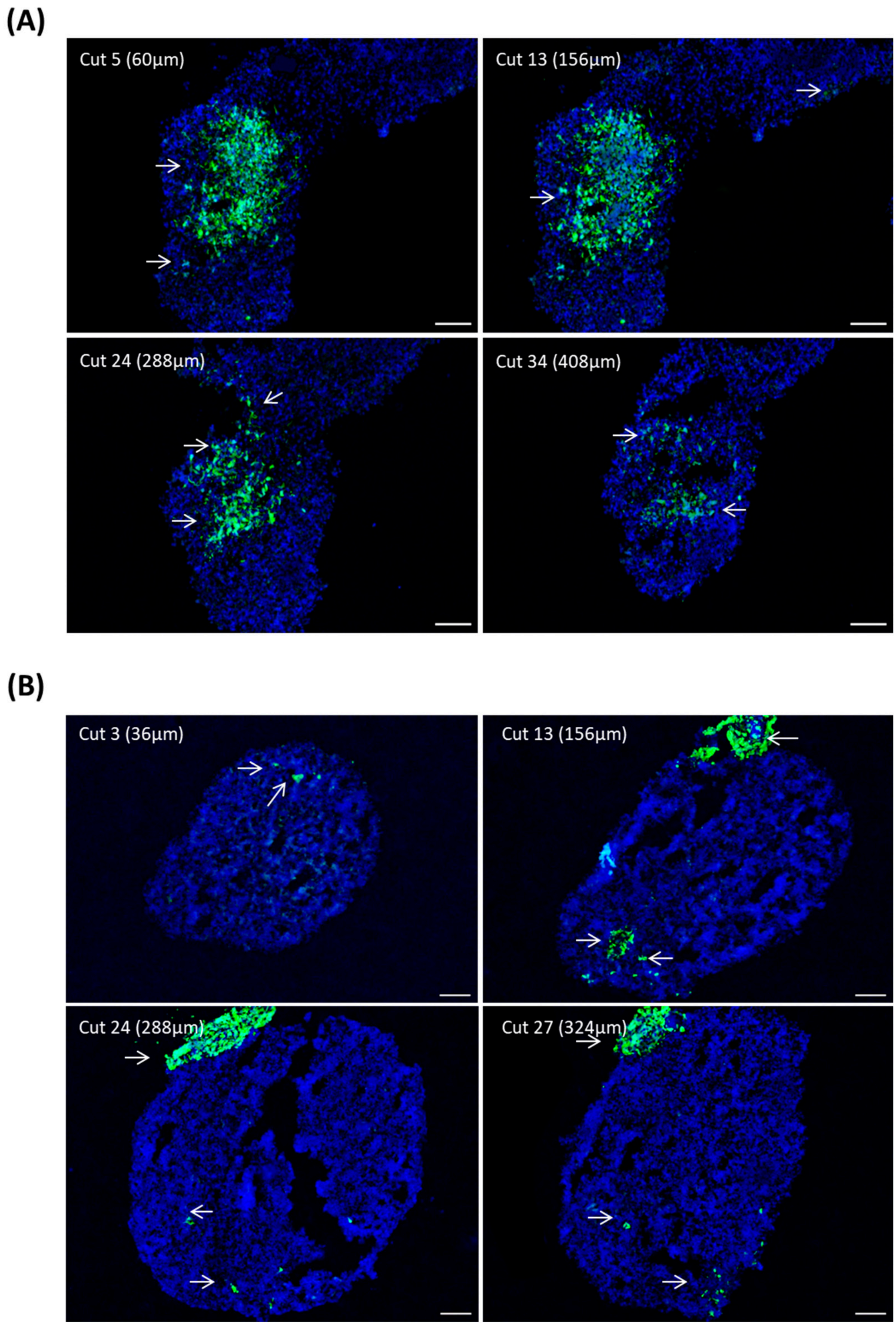

Figure S2. Invasive ability of GFP-GICs into organoids. Consecutive cuts in the same sample show infiltration of GFP-GIC7 (**A**) and GFP-PG88 (**B**) into the organoid at DoC 37. (**A**) For GFP-GIC7 the cut numbers 5, 13, 24, and 34 are shown, corresponding to 60, 156, 288, and 408 µm of depth into the organoid, respectively. (**B**) For GFP-PG88, the cut numbers 3, 13, 24, and 27 are shown, corresponding to 36, 156, 288, and 324 µm of depth into the organoid, respectively. The GFP-GICs are found in all cuts (white arrows)—nuclei labelled by Dapi. Scale bar 100 µm (10×).

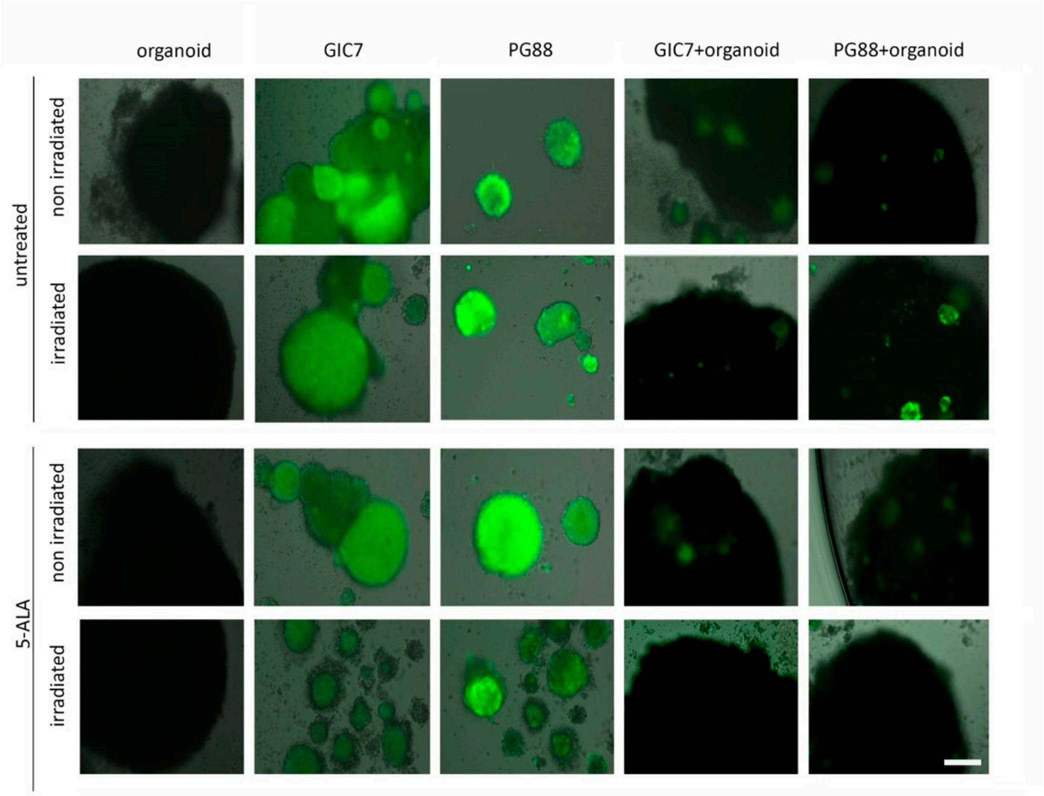

Figure S3. Comparison between co-cultures of brain organoids engrafted with GIC7 and PG88 GFP-GICs cells. Green fluorescence pictures of brain organoids and GICs taken 72 h after cells were exposed to the photodynamic treatment consisted on 50 μg/mL of 5-ALA incubated for 24 h. Pictures are representative of many pictures made in each of the plate wells. Scale bar: 250 μm.

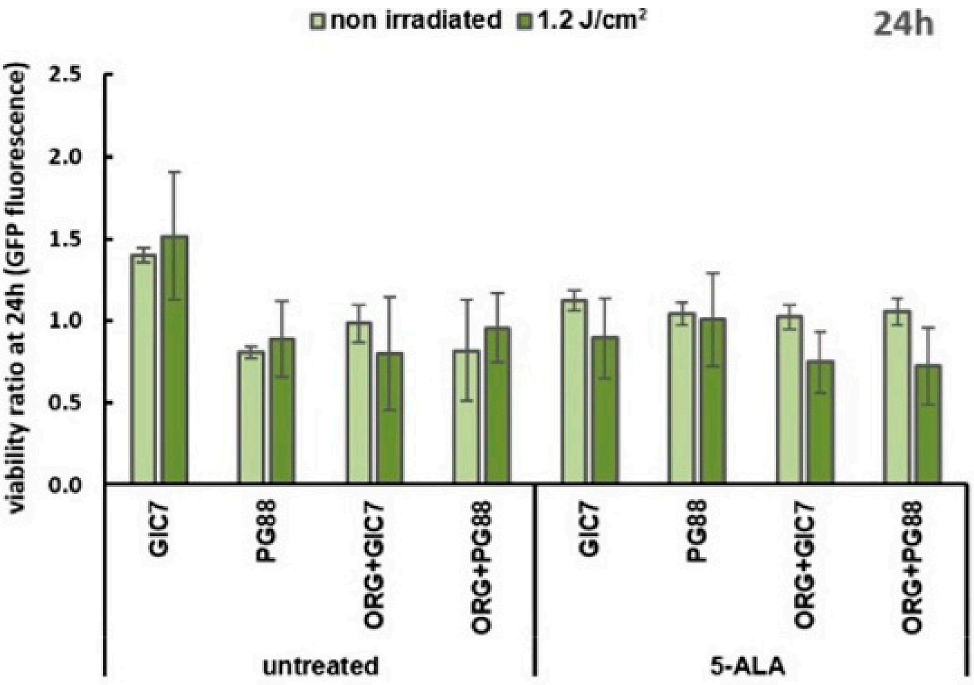

Figure S4. Cell viability after PDT of tumorspheres alone and co-cultured with brain organoids. GICs viability after 24 h post-irradiation using the GFP fluorescence ratio between each endpoint and time 0. Results are the mean ± SE of two independent experiments (n = 2) performed using six replicates.
